# Derivation and external validation of a simple prediction rule for the development of respiratory failure in hospitalized patients with influenza

**DOI:** 10.1186/s12931-022-02245-w

**Published:** 2022-11-24

**Authors:** Blanca Ayuso, Antonio Lalueza, Estibaliz Arrieta, Eva María Romay, Álvaro Marchán-López, María José García-País, Dolores Folgueira, María José Gude, Cecilia Cueto, Antonio Serrano, Carlos Lumbreras

**Affiliations:** 1 Department of Internal Medicine, University Hospital, 12 de Octubre, Av Córdoba Km 5,400, 28041 Madrid, Spain; 2 Infectious Diseases Unit, University Hospital Lucus Augusti, Lugo, Spain; 3 Department of Microbiology, University Hospital 12 de Octubre, Madrid, Spain; 4 Department of Microbiology, University Hospital Lucus Augusti, Lugo, Spain; 5 Department of Biochemistry, University Hospital 12 de Octubre, Madrid, Spain; 6 Department of Immunology, University Hospital 12 de Octubre, Madrid, Spain; 7 Infectious Diseases Unit, University Hospital 12 de Octubre, Madrid, Spain

**Keywords:** Influenza, Human, Pneumonia, Viral, Respiratory failure, Clinical prediction rules, Mechanical ventilation

## Abstract

**Background:**

Influenza viruses cause seasonal epidemics worldwide with a significant morbimortality burden. Clinical spectrum of Influenza is wide, being respiratory failure (RF) one of its most severe complications. This study aims to elaborate a clinical prediction rule of RF in hospitalized Influenza patients.

**Methods:**

A prospective cohort study was conducted during two consecutive Influenza seasons (December 2016–March 2017 and December 2017–April 2018) including hospitalized adults with confirmed A or B Influenza infection. A prediction rule was derived using logistic regression and recursive partitioning, followed by internal cross-validation. External validation was performed on a retrospective cohort in a different hospital between December 2018 and May 2019.

**Results:**

Overall, 707 patients were included in the derivation cohort and 285 in the validation cohort. RF rate was 6.8% and 11.6%, respectively. Chronic obstructive pulmonary disease, immunosuppression, radiological abnormalities, respiratory rate, lymphopenia, lactate dehydrogenase and C-reactive protein at admission were associated with RF. A four category-grouped seven point-score was derived including radiological abnormalities, lymphopenia, respiratory rate and lactate dehydrogenase. Final model area under the curve was 0.796 (0.714–0.877) in the derivation cohort and 0.773 (0.687–0.859) in the validation cohort (p < 0.001 in both cases). The predicted model showed an adequate fit with the observed results (Fisher’s test p > 0.43).

**Conclusion:**

we present a simple, discriminating, well-calibrated rule for an early prediction of the development of RF in hospitalized Influenza patients, with proper performance in an external validation cohort. This tool can be helpful in patient’s stratification during seasonal Influenza epidemics.

**Supplementary Information:**

The online version contains supplementary material available at 10.1186/s12931-022-02245-w.

## Introduction

Influenza epidemics relate to global mortality and morbidity each year, which entails a Public Health challenge. The net impact of an influenza epidemic results of the combination of the virus adaptability, its intrinsic virulence, and population susceptibility [1].

The actual burden of influenza epidemics is difficult to estimate due to the large variability in hospitalization and death reports [2]. A 2017 study reported an annual amount of 9 million influenza-related hospital admissions, more than 81 million hospitalization days, and almost 55 million respiratory tract infection episodes, from which 15% were severe [3]. A recent study estimated the annual death toll of influenza at almost 400,000 deaths, 2% of the total respiratory disease mortality [4].

Influenza disease spectrum ranges from mild cases with fever and malaise to severe pneumonia with respiratory failure (RF) and death [5]. RF development is unsteadily reported, due to heterogeneous definitions among available observational data. Some studies consider hypoxemia or a diminished blood partial pressure of oxygen (pO_2_) [6], while others define RF as the need for mechanical ventilation [7, 8], and so the rate of RF swings from 5% to more than 50% in hospitalized patients [9, 10].

Assessment of RF is more frequent in studies conducted during or immediately after the 2009 influenza pandemic [7, 9, 11], and in potentially pandemic avian influenza viruses like H5N1 and H7N9 [12, 13], and results may not match those from seasonal influenza.

Despite the importance of RF on the prognosis and impact of influenza, no tools have been developed to predict it. This study aims to develop and validate a clinical prediction rule (CPR) for the development of RF in patients hospitalized with influenza.

## Methods

### Study population and design

Development of the CPR was conducted on a prospective cohort involving two consecutive influenza seasons (December 2016 to March 2017, and December 2017 to April 2018) in a tertiary teaching hospital (University Hospital 12 de Octubre, Madrid, central Spain). External validation was undertaken on an ad hoc retrospective cohort from a different tertiary hospital (Lucus Augusti University Hospital, Lugo, northwestern Spain) from December 2018 to May 2019.

Patients older than 18 years with molecular biology-confirmed diagnosis of influenza virus infection who needed hospital admission for more than 24 h were considered for inclusion. Both A and B subtypes were included. Epidemiological, clinical, and therapeutical variables were collected, as well as laboratory parameters and radiology results at hospital admission. In order to increase the statistical power all the patients that were admitted to the hospital were included and no sample size was calculated beforehand.

For the development cohort, written informed consent was obtained for all patients. A waiver for informed consent was granted for the validation cohort. The study was approved by the Ethics Committees at 12 de Octubre University (reference 16/210 and 17/406) and at Lucus Augusti University Hospital (reference 2021/122).

### Molecular methods

In the development cohort, infection was confirmed with reverse transcription-polymerase chain reaction (rRT-PCR) using 118 LightCycler 480 (Roche, Rotkreuz, Switzerland). In the validation cohort, loop-mediated isothermal amplification (LAMP) with the Alere™ I Influenza A&B kit (Alere, Scarborough, ME, United States), was used.

### Definitions

The main outcome was the development of RF, defined as the necessity of mechanical ventilation (MV), either invasive mechanical ventilation (IMV) or non-invasive positive-pressure ventilation (NIPPV). Patients with ventilatory support indication who finally did not receive it due to comorbidities or performance status were considered as well as having RF.

Radiological abnormalities were defined as the presence of at least one infiltrate on chest radiograph on admission. Secondary pneumonia was defined as the suspected or confirmed presence of a bacterial superinfection during the influenza episode.

### Statistical analysis

TRIPOD recommendations were followed for the development and validation of CPR in this study [14]. Quantitative variables are reported as median and interquartile range; categorical variables with frequencies and percentages. Student’s T test and Wilcoxon-Mann–Whitney tests were used for quantitative variables, while Pearson’s Chi-squared test and Fisher’s exact test were used for categorical variables, as appropriate. Uni- and multivariate logistic regression, using backwards stepwise elimination in the latter case, and recursive partitioning via decision trees were used for predictive variable and cutoff values selection. Statistical significance was considered with p-values under 0.05. Statistical analysis was conducted using SPSS Statistics (version 25.0, IBM Corporation. Armonk, NY, United States).

### Derivation of prediction rules

A risk score using clinical, laboratory and radiography variables upon patient admission was derived. Predictor variables were selected using logistic regression. Multivariate logistic regression was conducted starting from a full model including every variable significantly associated with the outcome and other variables with biological relevance or a previously documented association with the outcome. After stepwise elimination, variables with a final p-value less than 0.05 and those with biological significance and a p-value under 0.10 remained in the model. A maximum of one variable every 10 events was considered. Multicollinearity was tested in the initial and final models to prevent overfitting.

Recursive partitioning was used to assign scores to continuous variables. Scores were assigned to categorical values based on their odds ratios in the final logistic model. Categories were built grouping scores with analogous risk of developing RF to simplify the use and interpretation of the tool results. Performance of the final and intermediate models was assessed using sensitivity, specificity, positive and negative predictive values, and overall accuracy, as well as visually using receiver operating characteristic (ROC) curves and their area under the curve (AUC) with its 95% confidence interval and statistical significance. The model was manually tuned to maximize its discrimination power while keeping it the simplest. A risk model of RF development in the different categories was elaborated using bootstrapping with 1000 replicates.

### Missing values

Variables with more than 10% of missing values which could not be considered as missing completely at random were imputed generating five additional data sets.

### Validation

Internal validation was conducted using five-fold cross validation. Cases in the original set were shuffled, then the sample was divided in five subsets. Analysis of the model performance was repeated five times removing one subset each time.

External validation was conducted by calculating the risk score for every patient and assigning them into the different categories. The observed RF by risk category distribution was compared with the distribution predicted by the model using Pearson’s Chi-squared test for goodness-of-fit and Fisher’s exact test.

## Results

### Sample characteristics

**Fig. 1:**
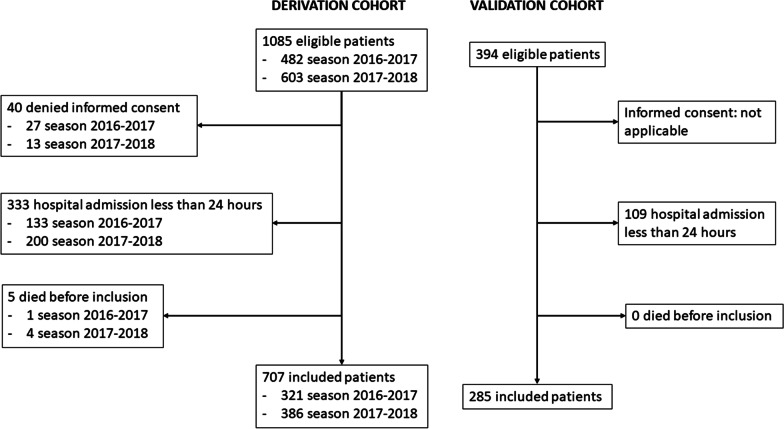
Patient inclusion flowchart

A total of 1085 influenza virus infections were diagnosed in adults during the development stage of this study, 482 in the influenza season of 2016–2017, and 603 in the 2017–2018 season. After inclusion and exclusion criteria were applied, 707 patients remained (Fig. 1). Influenza A was the most frequent subtype (561/707, 79.3%), followed by influenza B (145/707, 20.5%), while only one patient (0.01%) had influenza A and B coinfection.

Three hundred ninety-four infections were diagnosed in the validation stage, with 285 patients finally included in the external validation cohort.

Patients’ characteristics are reported in Table 1. In the derivation cohort, median age was 79 (65–85) years, 52.6% of patients were male, and comorbidity as moderate (median Charlson Comorbidity Index 2, 1–5). The median of oxygen saturation (SpO_2_) on room air at admission was 92% (88–95) and median respiratory rate was 18 (16–24) breaths per minute. Radiological abnormalities were present in 40.5% of patients, and the median lymphocyte count was 700 cells/µL (400–1000). Forty-eight patients (6.8%) developed RF during hospitalization. From all patients, 4.2% were admitted to an intensive care unit (ICU) irrespective of the reason, 2.5% received NIPPV, and 2.7% received IMV. 43.8% of the patients with RF needed ICU admission and 62.5% received ventilatory support. Thirty-four patients (4.8%) died during hospitalization.

**Table 1:** General characteristics of the samples

Variable, n (%)	Total (N = 992)	Derivation cohort (n = 707)	Validation cohort (n = 285)	p
Influenza subtype				
A	846 (85.3%)	561 (79.3%)	285 (100.0%)	< 0.001
B	145 (14.5%)	145 (20.5%)	0 (0.0%)	
Both	1 (0.1%)	1 (0.1%)	0 (0.0%)	
Age (median, IQR)	79 (66–85)	79 (65–85)	79 (67–85)	> 0.20
Sex (male)	522 (52.6%)	372 (52.6%)	150 (52.6%)	> 0.20
Days since symptoms (median, IQR)	3 (2–5)	3 (2–6)	3 (1–4)	< 0.001
Symptoms lasting > 72 h	427 (43.6%)	327 (47.1%)	100 (35.1%)	0.001
Influenza vaccine	480 (48.4%)	325 (46.0%)	155 (54.4%)	0.017
Hypertension	672 (67.7%)	484 (68.5%)	188 (66.0%)	> 0.20
Pregnancy	9 (0.9%)	7 (1.0%)	2 (0.7%)	> 0.20
Current smokers	126 (12.8%)	91 (12.9%)	35 (12.7%)	> 0.20
Obesity	196 (45.6%)	108 (46.0%)	88 (45.1%)	> 0.20
Asthma	90 (9.1%)	73 (10.3%)	17 (6.0%)	0.03
COPD	242 (24.4%)	172 (24.3%)	70 (24.6%)	> 0.20
Diabetes mellitus	276 (27.8%)	206 (29.1%)	70 (24.6%)	0.15
Cardiovascular disease	348 (35.1%)	240 (33.9%)	108 (37.9%)	> 0.20
Chronic kindney disease	176 (17.7%)	127 (18.0%)	49 (17.2%)	> 0.20
Liver disease	53 (5.3%)	36 (5.1%)	17 (6.0%)	> 0.20
Neurologic disorder	170 (17.1%)	188 (16.7%)	52 (18.2%)	> 0.20
Immunosupression (*)	177 (17.8%)	132 (18.7%)	45 (15.8%)	> 0.20
CCI (median. IQR)	2 (1–4)	2 (1–5)	2 (1–3)	< 0.001
CCI > 2 points	620 (62.8%)	458 (65.2%)	162 (56.8%)	0.01
Respiratory rate (median, IQR)	20 (16–25)	18 (16–24)	22 (16–28)	< 0.001
pO2 on room air (median, IQR)	56 (50–64)	57 (50–65)	54 (50–61)	0.004
SpO2 on room air (median, IQR)	91 (88–95)	92 (88–95)	91 (87–95)	0.099
SpO2 on room air < 94%	627 (66.6%)	440 (66.4%)	187 (67.0%)	> 0.20
Radiological abnormalities	396 (41.7%)	274 (40.5%)	122 (44.9%)	> 0.20
Secondary pneumonia	161 (16.4%)	135 (19.1%)	26 (9.5%)	< 0.001
Lymphocyte count (cells/µl) (median, IQR)	700 (400–1000)	700 (400–1000)	700 (400–1000)	> 0.20
LDH (U/l) (median, IQR)	275 (224–335)	287 (246–348)	228 (188–292)	< 0.001
Respiratory failure	81 (8.2%)	48 (6.8%)	33 (11.6%)	0.01
Admission to ICU	49 (4.9%)	30 (4.2%)	19 (6.7%)	0.11
Mechanical ventilation				
NIPPV	36 (3.6%)	18 (2.5%)	18 (6.3%)	0.004
IMV	22 (2.2%)	19 (2.7%)	3 (1.1%)	0.11
Mortality				
In hospital (overall)	47 (4.8%)	33 (4.7%)	14 (4.9%)	> 0.20
30-day	25 (2.6%)	18 (2.5%)	7 (2.6%)	> 0.20
Evolution after discharge				
Recovery without sequelae	794 (80.3%)	592 (84.1%)	202 (70.9%)	< 0.001
Recovery with sequelae	148 (15.0%)	79 (11.2%)	69 (24.2%)	
Length of hospital stay (days. median. IQR)	7 (5–13)	7 (5–12)	8 (5–16)	0.013

*COPD* chronic obstructive pulmonary disease, *CCI* Charlson Comorbidity Index, *pO*_*2*_ partial pressure of oxygen, *SpO*_*2*_ oxygen saturation, *LDH* lactacte dehydrogenase, *ICU* Intesive Care Unit, *NIPPV* non-invasive positive pressure ventilation, *IMV* invasive mechanical ventilation

*Immunosuppression was defined as the presence of any of: active cancer, autoimmune disease, solid organ or hematological transplantation, HIV infection, treatment with immunosuppressive drugs or active chemotherapy (15)

In the external validation cohort, all influenza isolates corresponded to influenza A. Median duration of symptoms before hospital admission was shorter [3 (1–4) days *vs* 3 (2–6) days, p < 0.001], and more patients were vaccinated (54.5% *vs* 46.0%, p = 0.017) in the validation cohort. Radiological findings were similar (44.9% *vs* 40.5%, p > 0.20), but fewer secondary pneumonia episodes were observed in the second cohort (9.5% *vs* 19.1%, p < 0.001). Thirty-three patients developed RF in this cohort (11.6% *vs* 6.8%, p = 0.013). No differences were observed in mortality or ICU admissions, but more patients received NIPPV (6.3% *vs* 2.5%, p = 0.004) in the validation cohort.

### Predictive model

Variables included into the multivariate logistic regression model and those remaining after backwards stepwise elimination are reported in Table 2. Lymphocytes at admission were kept in on grounds of prognostic implications in the extant literature and a statistical significance of p < 0.10.

**Table 2:** Univariate and multivariate logistic regression analysis

VARIABLE	Univariate OR (CI 95%)	p	Multivariate OR (CI 95%)	p	FM OR (CI 95%)	p
Age*	0.980 (0.964–0.997)	0.023	0.977 (0.955–0.999)	0.037		
Sex (male)	1.339 (0.744–2.410)	0.331				
Influenza vaccine	0.623 (0.338–1.148)	0.129				
Hypertension	0.753 (0.410–1.381)	0.359				
Current smokers	1.624 (0.759–3.475)	0.212				
Obesity	0.610 (0.254–1.467)	0.270				
COPD	1.783 (0.961–3.309)	0.067	1.980 (0.954–4.109)	0.067		
Diabetes mellitus	0.622 (0.304–1.272)	0.193				
Immunossupresion	0.604 (0.251–1.453)	0.260	0.671 (0.265–1.704)	0.671		
CCI	1.030 (0.922–1.151)	0.599				
Respiratory rate*	1.114 (1.090–1.200)	< 0.001	1.112 (1.055–1.172)	< 0.001	1.110 (1.056–1.168)	0.000
SpO_2_ on room air*	0.927 (0.895–0.960)	< 0.001				
Radiological abnormalities	2.093 (1.149–3.814)	0.016	2.826 (1.333–5.991)	0.007	2.715 (1.341–5.498)	0.006
Secondary pneumonia	3.067 (1.663–5.657)	< 0.001				
Platelets (cells/µl)*	1.002 (0.999–1.005)	0.224				
Lymphocytes (cells/µl)*	1.008 (0.995–1.021)	0.217	1.000 (1.000–1.000)	0.070	1.000 (1.000–1.000)	0.094
CRP (mg/dl)*	1.020 (0.992–1.049)	0.160	1.002 (0.968–1.037)	0.929		
LDH (U/l)*	1.004 (1.002–1.005)	< 0.001	1.003 (1.001–1.005)	0.002	1.003 (1.002–1.005)	0.000

*OR* odds ratio, *CI* confidence interval, *FM* final multivariate model after backwards elimination, *COPD* chronic obstructive pulmonary disease, *CCI* Charlson Comorbidity Index, *SpO*_*2*_ oxygen saturation, *CRP* C-reactive protein, *LDH* lactate dehydrogenase. *Odds Ratio per increment of 1 unit

### Missing value imputation

The only variable with more than 10% missing values from those included in the models was respiratory rate (245 missing). Missing respiratory rate values were imputed five times using multiple imputation, resulting in six sets of data. Statistical analysis and performance assessment was conducted once in each set.

### Derivation of clinical prediction rules

**Fig. 2:**
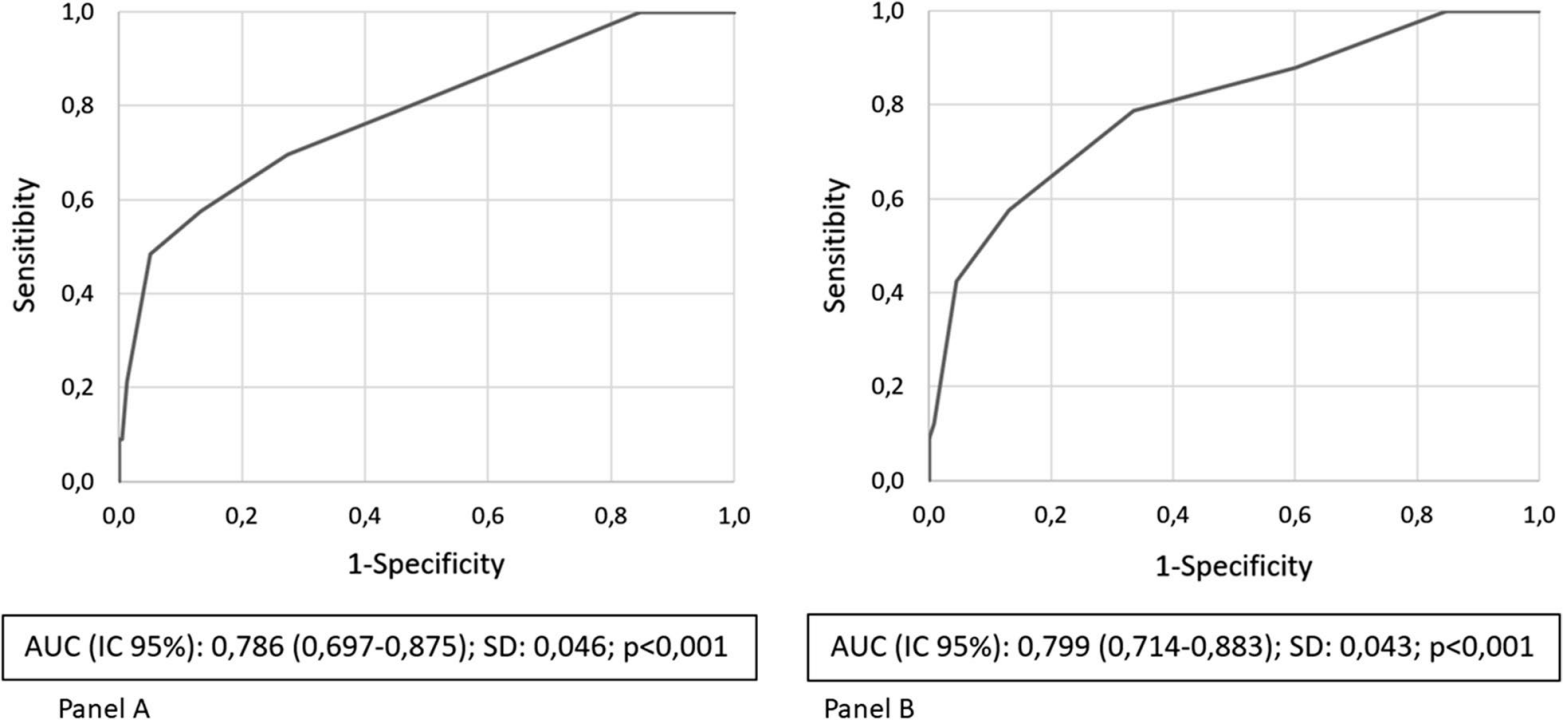
Derivation cohort risk scores receiver operating characteristic (ROC) curves. **A** original score (9 points). **B** simplified score (7 points). *AUC* area under curve, *SD* standard deviation

A formula for RF prediction was created using coefficients of the final logistic regression model but, due to the predicted event being infrequent, low sensitivity was achieved. Since the aim of the study was to develop a scale of risk categories, recursive partitioning was used to ascribe risk scores. Decision trees were performed separately for each variable since no interaction or collinearity was found. Scores were assigned according to the RF proportion in the different intervals of continuous variables: zero points assigned to intervals with less than 5% RF, one point assigned to intervals with 5–10% RF, and two points assigned to intervals with more than 10% RF. Two points were assigned to patients with radiological abnormalities considering and Odds Ratio of 2 in the multivariate logistic regression. The score was manually fine-tuned using ROC curves, and simplified to a maximum possible score of 7 points, and scores were grouped into four categories (Fig. 2).

The final risk score is computed by assigning 0–2 points to the lymphocyte count at admission, 0–2 points to the lactate-dehydrogenase (LDH) levels at admission, 0–2 points to the respiratory rate at admission, and 1 point if radiological abnormalities are present. This score is then converted to risk categories, with a zero-point score corresponding to category A, an one- or two-point score corresponding to category B, a three- or four-point score to category C, and a five-point score or higher to category D. RF risk was computed for each category using 1000 sample simulation bootstrapping (Table 3). Precision analysis is reported in Additional file 1: appendix 1.

**Table 3:** Risk score and risk categories

Variable	Score
Lymphocytes at admission	
≥ 600 cells/µl	0 point
400–600 cells/µl	1 point
≤ 400 cells/µl	2 points
Respiratory rate	
≤ 20 brpm	0 point
21–28 brpm	1 point
> 28 brpm	2 points
LDH at admission	
≤ 280 U/l	0 point
280–400 U/l	1 point
> 400 U/l	2 points
Radiological alteration	
No	0 point
Yes	1 point

Risk category	Respiratory failure proportion (CI 95%)
A: 0 point	0.0% (0.0–0.0%)
B: 1–2 points	3.5% (1.0–6.6%)
C: 3–4 points	10.0% (5.0–15.8%)
D: 5 or more points	45.2% (29.0–61.3%)

*brpm* breath per minute, *CI* confidence interval

### Validation of the clinical prediction rule

Internal validation was performed using five-fold cross-validation. The clinical prediction rule maintained its discrimination capacity in the five subsets within the derivation cohort (Additional file 2: appendix 2).

**Fig. 3:**
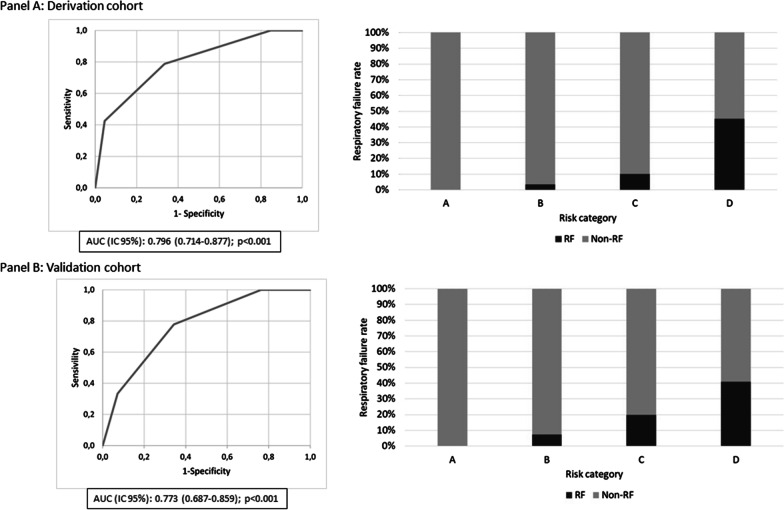
receiver operating characteristic (ROC) curves for risk categories and respiratory failure distribution in both cohorts. **A** Derivation cohort. **B** Validation cohort. *AUC* area under the curve, *RF* respiratory failure

Risk scores and categories were computed for patients in the external validation cohort. ROC curves for both cohorts are shown in Fig. 3. Area under the ROC curve was 0.796 (0.714–0.877) in the derivation cohort and 0.773 (0.687–0.859) in the validation cohort (p-value < 0.001 in both cases). Convenient classification power was observed in both cohorts, with a conclusive Chi-squared test for trend for risk categories and RF development (p-value < 0.05 in all cases). An adequate fit was observed between predicted and observed RF proportions in the four categories (Fisher’s exact test for goodness-of-fit p-value = 0.43; Chi-squared test for goodness-of-fit with merging of 0 predicted event categories p-value = 0.42).

## Discussion

Classification of influenza patients in terms of RF development probabilities upon admission could allow for better management of resources and a tailored care provision. Patients with insignificant risk could be safely discharged early in peak incidence settings, avoiding bottlenecking and collapse of healthcare institutions. The development of a tool with these characteristics would be more significant since major pneumonia severity scales perform poorly in influenza [16, 17].

To our knowledge, no other respiratory failure prediction tools have been communicated. An approach to this issue was conducted by Oh et al., who developed a prediction rule considering a composite outcome of death, mechanical ventilation, and ICU admission, including mental status alteration, oxygenation index, bilateral radiographic involvement, and age [18], with a slightly higher specificity but lower sensitivity compared to the one we present. Other tools with similar performance have been proposed for community acquired pneumonia [19] or COVID-19 [20].

Patients admitted with influenza are often aged and have several comorbidities, which prevents them from receiving ventilatory support on account of likely futility, and eventually leads to withholding or withdrawing life-sustaining treatments. The practical RF definition used in this study allows the tool to be used in those patients, making it useful in the usual clinical practice.

Variables included in our CPR have been previously associated with adverse outcomes in influenza. Hematological abnormalities, especially lymphopenia, have been associated with a poor prognosis in influenza infection [21–23]. High LDH levels have been related to worse outcomes in influenza virus infection [24] and other respiratory viruses like SARS-CoV-2 [25]. Respiratory rate is already used in tools like the National Early Warning Score (NEWS) 2 [26].

We found an inverse relationship between age and RF development. This could be explained since younger patients with influenza are usually admitted only when they portrait a severe clinical picture or when they have other risk factors. In the derivation cohort, patients older than 80 years had a higher death risk even in the absence of respiratory failure.

There are some remarkable differences between our two cohorts. A higher rate of RF is observed in the validation cohort. This could be explained since patients in that cohort presented a more severe clinical picture, with lower SpO_2_, higher respiratory rates, and a trend towards a higher rate of primary pneumonia, despite not having more comorbidities nor being less vaccinated. This in turn might be justified by a different threshold for hospital admissions or the different circulating serotypes in the two cohorts: there were no influenza B isolates in the validation cohort. In the 2018–2019 season in Spain, influenza A was by far the dominant serotype, with 0.43% of cases of influenza being caused by serotype B [27].

The higher rate of secondary pneumonia observed in the derivation cohort can be partially explained because urinary pneumococcal antigen detection test kits were only available in the validation cohort setting, allowing to rule out superinfection more easily. It is also notable that respiratory rate was more often missing in the derivation cohort, while it was a requisite in the Emergency Department admission forms in the validation institution.

The main strength in this study is the performance of the prediction rule that we present. It is a simple, parsimonious tool, with an adequate classification power, which reports results in the shape of intuitive, distinct categories. It also performs properly across different RF prevalences and in cohorts with different basal characteristics and case management (i.e. use of NIPPV).

The retrospective acquisition of the validation cohort data might affect the sensitivity of the results, which represents a limitation of our study. Although missing values could imply a limitation in the derivation of the rule, replacement via multiple imputation did not alter the performance of the rule when applied to the validation cohort.

In conclusion, we propose a simple but effective tool for an early stratification of hospitalized patients with influenza according to their risk of RF. This risk score demonstrates an adequate performance in two cohorts with different RF incidences and management. In the future, it would be interesting to assess the performance of our tool in further cohorts of influenza as well as in other respiratory infections, including those with pandemic potential.

## Supplementary Information

**Additional file 1: Appendix 1.** score precision analysis. Sensitivity, specificity, predictive values and accuracy of the different risk categories in the two cohorts.

**Additional file 2: Appendix 2.** internal validation. Five-fold cross validation of the score; Chi squared and Chi squared test for trend results are showed for the five subsets.

## Data Availability

Anonymized clinical data sets are available upon corresponding author contact.
