# Supplementary material for: Derivation and external validation of a simple prediction rule for the development of respiratory failure in hospitalized patients with influenza
Source: Respir Res. 2022 Nov 24;23:323. doi: 10.1186/s12931-022-02245-w (PMC9684757; doi:10.1186/s12931-022-02245-w)
Supplement: Supplementary file 1 — Additional file 1: Appendix 1. score precision analysis. Sensitivity, specificity, predictive values and accuracy of the different risk categories in the two cohorts. [file 12931_2022_2245_MOESM1_ESM.docx]

**Appendix 1: score precision analysis.**

Panel A: Derivation cohort

| CATEGORY | SE (CI 95%) | SP (CI 95%) | PPV (CI 95%) | NPV (CI 95%) | A (CI 95%) |
| --- | --- | --- | --- | --- | --- |
| B or greater | 100,0 (89,4-100,0) | 15,3 (11,8-19,3) | 9,5 (9,1-9,8) | 100,0 | 22,2 (18,2-26,5) |
| C or greater | 78,8 (61,1-91,0) | 66,5 (61,5-71,3) | 17,2 (14,2-20,7) | 97,2 (94,8-98,6) | 67,5 (62,7-72,0) |
| D | 42,4 (25,5-60,8) | 95,4 (92,8-97,3) | 45,2 (30,9-60,3) | 94,9 (93,3-96,2) | 91,1 (87,9-93,7) |

Panel B: Validation cohort.

| CATEGORY | S (CI 95%) | E (CI 95%) | PPV (CI 95%) | NPV (CI 95%) | A (CI 95%) |
| --- | --- | --- | --- | --- | --- |
| B or greater | 100,0 (87,3-100,0) | 23,8 (17,8-30,6) | 16,4 (15,3-18,3) | 100,0 | 33,6 (27,3-40,5) |
| C or greater | 77,8 (57,7-91,4) | 65,8 (58,3-72,6) | 25,3 (20,3-31,1) | 95,2 (90,7-97,6) | 67,3 (60,5-73,6) |
| D | 33,3 (16,5-54,0) | 92,8 (88,0-96,1) | 40,9 (24,7-59,4) | 90,3 (87,7-92,4) | 85,1 (79,5-89,6) |

SE: sensitivity; SP: specificity; PPV: positive predictive value; NPV: negative predictive value; A: accuracy.

Category A: 0 points; B: 1-2 points. C: 3-4 points. D: 5 or more points.
