# Supplementary material for: Derivation and external validation of a simple prediction rule for the development of respiratory failure in hospitalized patients with influenza
Source: Respir Res. 2022 Nov 24;23:323. doi: 10.1186/s12931-022-02245-w (PMC9684757; doi:10.1186/s12931-022-02245-w)
Supplement: Supplementary file 2 — Additional file 2: Appendix 2. internal validation. Five-fold cross validation of the score; Chi squared and Chi squared test for trend results are showed for the five subsets. [file 12931_2022_2245_MOESM2_ESM.docx]

**Appendix 2: internal validation method.**

| Subset | Category | Respiratory failure proportion (%) |
| --- | --- | --- |
| 1 | A | 0% |
|  | B | 3.1% |
|  | C | 10.5% |
|  | D | 44.8% |
|  | χ2 = 59.5; p<0.001. χ2 for trend = 41.5; p<0.001 | |
| 2 | A | 0% |
|  | B | 3.8% |
|  | C | 9.2% |
|  | D | 37.5% |
|  | χ2 =39.4; p<0.001. χ2 for trend = 27.2; p<0.001. | |
| 3 | A | 0% |
|  | B | 4.5% |
|  | C | 10.8% |
|  | D | 52.2% |
|  | χ2 = 60.2; p<0.001. χ2 for trend = 38.2; p<0.001. | |
| 4 | A | 0% |
|  | B | 3.8% |
|  | C | 9.4% |
|  | D | 47.8% |
|  | χ2 = 57.8; p<0.001. χ2 for trend = 35.8; p<0.001. | |
| 5 | A | 0% |
|  | B | 2.5% |
|  | C | 10.2% |
|  | D | 44.0% |
|  | χ2 = 57.1; p<0.001. χ2 for trend = 39.0; p<0.001. | |
